# Disabling de novo DNA methylation in embryonic stem cells allows an illegitimate fate trajectory

**DOI:** 10.1073/pnas.2109475118

**Published:** 2021-09-13

**Authors:** Masaki Kinoshita, Meng Amy Li, Michael Barber, William Mansfield, Sabine Dietmann, Austin Smith

**Affiliations:** ^a^Wellcome-MRC Cambridge Stem Cell Institute, Jeffrey Cheah Biomedical Centre, University of Cambridge, Cambridge CB2 0AW, United Kingdom;; ^b^Department of Biochemistry, University of Cambridge, Cambridge CB2 1GA, United Kingdom;; ^c^Living Systems Institute, University of Exeter, Exeter EX4 4QD, United Kingdom

**Keywords:** DNA methylation, embryonic stem cells, pluripotency

## Abstract

Genome remethylation is essential for mammalian development but specific reasons are unclear. Here we examined embryonic stem (ES) cell fate in the absence of de novo DNA methyltransferases. We observed that ES cells deficient for both *Dnmt3a* and *Dnmt3b* are rapidly eliminated from chimeras. On further investigation we found that in vivo and in vitro the formative pluripotency transition is derailed toward production of trophoblast. This aberrant trajectory is associated with failure to suppress activation of *Ascl2*. *Ascl2* encodes a bHLH transcription factor expressed in the placenta. Misexpression of *Ascl2* in ES cells provokes transdifferentiation to trophoblast-like cells. Conversely, *Ascl2* deletion rescues formative transition of *Dnmt3a/b* mutants and improves contribution to chimeric epiblast. Thus, de novo DNA methylation safeguards against ectopic activation of *Ascl2*. However, *Dnmt3a/b*-deficient cells remain defective in ongoing embryogenesis. We surmise that multiple developmental transitions may be secured by DNA methylation silencing potentially disruptive genes.

## Significance

Mammalian DNA is widely modified by methylation of cytosine residues. This modification is added to DNA during early development. If methylation is prevented, the embryo dies by midgestation with multiple abnormalities. In this study we found that stem cells lacking the DNA methylation enzymes do not differentiate efficiently into cell types of the embryo and are diverted into producing placental cells. This switch in cell fate is driven by a transcription factor, Ascl2, which should only be produced in the placenta. In the absence of DNA methylation, the *Ascl2* gene is misexpressed. Removing Ascl2 redirects embryonic fate but not full differentiation potential, suggesting that methylation acts at multiple developmental transitions to restrict activation of disruptive genes.

The mammalian genome is characterized by widespread methylation of cytosine residues. After fertilization, however, both maternal and paternal genomes undergo extensive demethylation, reaching a low point in the blastocyst (1–4). The embryo genome is then remethylated by the activity of de novo DNA methylation enzymes (5). Mouse embryonic stem (ES) cells exhibit global hypomethylation, similar to the in vivo blastocyst profile (6–8). Methylation is gained during the formative pluripotency transition to lineage competence, recapitulating early postimplantation development in vivo (9, 10).

Mammals have three DNA methyltransferases (DNMTs). Dnmt1 maintains methylation during DNA replication, while Dnmt3a and Dnmt3b are responsible for de novo methylation. DNA methylation is not required for general cell viability and, with the exception of imprint control regions, largely occurs at seemingly nonfunctional regions of the genome (11). Nonetheless, knockout of *Dnmt1* in mice results in embryonic lethality around embryonic day 9.5 (E9.5) (12). *Dnmt3a* mutants die during puberty, but *Dnmt3b* mutant embryos fail from E9.5 onwards, exhibiting multiple abnormalities (13). When both de novo DNMTs are inactivated, development is disrupted by E8.5 with defective somitogenesis and abnormal morphogenesis. Seemingly normal progress to late gastrulation suggests that remethylation in the early postimplantation embryo is not critical for epiblast transition or germ layer formation. However, the reason(s) for the subsequent catastrophic failure is unclear. It has also been found that ES cells doubly deficient for *Dnmt3a* and *Dnmt3b* show a progressive genome-wide reduction in DNA methylation and loss of ability to form teratomas after long-term culture (14).

We previously showed that depletion of Dnmt3a and Dnmt3b delays timely exit from naive pluripotency in vitro (15). Here we investigate the functional consequences of the lack of de novo methylation in ES cells for pluripotency progression and lineage potential at the cellular level.

## Results

### Chimera Colonization and Lineage Potential of Dnmt3a/3b-Deficient ES Cells.

**Fig. 1.**
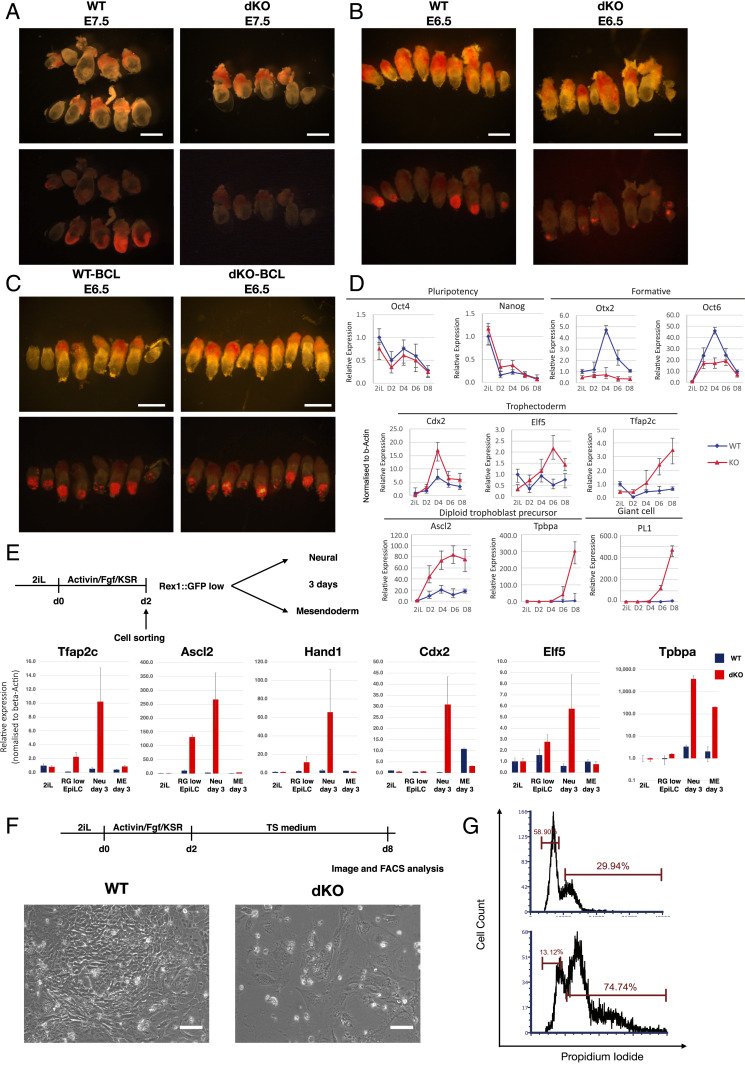
*Dnmt3a/b* deficiency compromises chimera contribution and somatic lineage commitment. (*A*–*C*) Chimeric embryos obtained with parental, Dnmt3dKO (*A* and *B*), and hBCL2-expressing (*C*) mKO2 reporter ES cells. The 30% opacity brightfield images are overlaid onto fluorescent images. (Scale bars, 1 mm in *A* and 500 µm in *B* and *C.*) (*D*) qRT-PCR analysis of marker during culture in trophoblast medium (20). (*E*) qRT-PCR analysis of undifferentiated ES cells, Rex1::GFP^Lo^ sorted AFK cells, and further differentiated cells. (*F*) Cell morphology of WT and Dnmt3dKO cells in trophoblast stem cell (TS) cell medium (20) for 8 d. (Scale bars, 50 µm.) (*G*) DNA content quantified by propidium iodide (PI) staining at day 8 in TS cell medium. qRT-PCR data were normalized to beta-actin. Error bars are SD from technical duplicates.

We examined the ability of *Dnmt3a* and *Dnmt3b* double knockout (Dnmt3dKO) ES cells (15) to contribute to chimeric embryos. Compound *Dnmt3a/3b* mutant embryos are reportedly normal until somitogenesis (13). However, after blastocyst injection of Dnmt3dKO cells genetically labeled with constitutive mKO2, we found very sparse contributions in presomite-stage embryos at E7.5 (Fig. 1*A*). Even at E6.5 contributions were reduced compared with typical ES cell chimeras (Fig. 1*B*). Furthermore, some mutant donor cells were located in the extraembryonic ectoderm, a rare occurrence with wild-type (WT) ES cells (16).

To improve survival of Dnmt3dKO ES cells in chimeras, we introduced a constitutive *BCL2* transgene (17). In E6.5 embryos we observed higher contributions to epiblast, comparable to wild-type ES cells (Fig. 1*C*). However, we also saw donor cells in extraembryonic regions. We confirmed localization in the extraembryonic ectoderm and ectoplacental cone in five out of six chimeras examined by confocal microscopy (*SI Appendix*, Fig. S1*A*). We inspected blastocyst-stage chimeras to test whether *Dnmt3a/b* deficiency or *BCL2* expression enabled primary trophectoderm colonization. However, donor cells were correctly localized to the inner cell mass (ICM) and did not contribute to trophectoderm (*SI Appendix*, Fig. S1*B*). Thus, the presence of Dnmt3dKO cells in postimplantation trophoblast likely arises by displacement from the epiblast rather than differentiation via trophectoderm.

The poor and aberrant colonization behavior of Dnmt3dKO cells prompted us to investigate in vitro differentiation competence. In response to mesendoderm induction, mutant cells up-regulated *T* and *Foxa2* but to a lower level than parental cells (*SI Appendix*, Fig. S1*C*). In permissive conditions for neural induction (18), Dnmt3dKO cells showed only weak up-regulation of *Sox1* and *Pax6* (*SI Appendix*, Fig. S1*D*) but displayed substantial and sustained up-regulation of *Ascl2*, *Hand1*, and *Tpbpa*, genes associated with the trophoblast lineage (*SI Appendix*, Fig. S1 *C* and *D*). To assess whether Dnmt3dKO cells adopt trophoblast identity, we applied two culture conditions for trophoblast cells (19, 20). Unlike parental cells, Dnmt3dKO cells showed no or low up-regulation of formative pluripotency factors Otx2 and Oct6, but instead gained expression of trophoblast markers (Fig. 1*D* and *SI Appendix*, Fig. S1*E*).

We also subjected single *Dnmt* KO ES cells (15) to lineage induction (*SI Appendix*, Fig. S1*F*). Expression of germ layer markers was reduced in both mutants, and in neural conditions trophoblast genes were up-regulated (*SI Appendix*, Fig. S1*F*). The phenotype was more marked in *Dnmt3b* KO cells, with higher trophoblast gene induction and lower neural and mesendodermal gene activation. We introduced expression constructs for *Dnmt3a* and *Dnmt3b* into Dnmt3dKO cells (*SI Appendix*, Fig. S1*G*). Rescued cells displayed normal differentiation with suppression of trophoblast genes (*SI Appendix*, Fig. S1*H*).

Culture in activin, FGF2, and KSR (AFK) induces ES cell conversion into postimplantation formative epiblast-like cells (EpiLCs) (21). Dnmt3dKO cells showed delayed morphological change on day 1 but by day 2 appeared similar to parental cells with a comparable increase in cell number (*SI Appendix*, Fig. S1 *I* and *J*). The Rex1::GFPd2 (RGd2) reporter allows reliable monitoring of ES cell exit from naive pluripotency (9). Dnmt3dKO cells showed delayed down-regulation of the reporter in AFK (*SI Appendix*, Fig. S1*K*), consistent with findings in N2B27 (15). We sorted the GFP low (GFP^Lo^) fraction that has exited naive pluripotency and saw that trophoblast genes *Ascl2* and *Tfap2c* were misexpressed in mutant cells (Fig. 1*E*). Levels increased further upon continued culture in N2B27 (Fig. 1*E*) while neural markers, *Sox1* and *Pax6*, were very lowly expressed (*SI Appendix*, Fig. S1*L*). GFP^Lo^ cells transferred into activin and CH gained only modest up-regulation of *T* and FoxA2, though they did not express most trophoblast markers.

After ongoing culture of dKO cells in N2B27, we observed additional trophoblast markers. Strikingly, however, the sequence of marker appearance differed from the in vivo developmental program. *Ascl2* and *Hand1*, evident after 48 h in AFK, are characteristic of postimplantation trophoblast, whereas *Cdx2* and *Elf5*, associated with primary trophectoderm, showed appreciable expression only at later stages (Fig. 1*E*). These results also differ from *Dnmt1* mutants where up-regulation of *Elf5* is thought to drive trophoblast transdifferentiation (22).

Finally, we transferred Dnmt3dKO cells from AFK into trophoblast medium (20). In contrast to parental cells, mutant cells flattened and some became very large with prominent nuclei (Fig. 1*F*). Propidium iodide staining showed a fraction with greater than 4N DNA content, consistent with polyploid trophoblast giant cell formation (Fig. 1*G*).

### Transcriptome Analyses of ES Cell Differentiation Trajectory in the Absence of Dnmt3a/b.

**Fig. 2.**
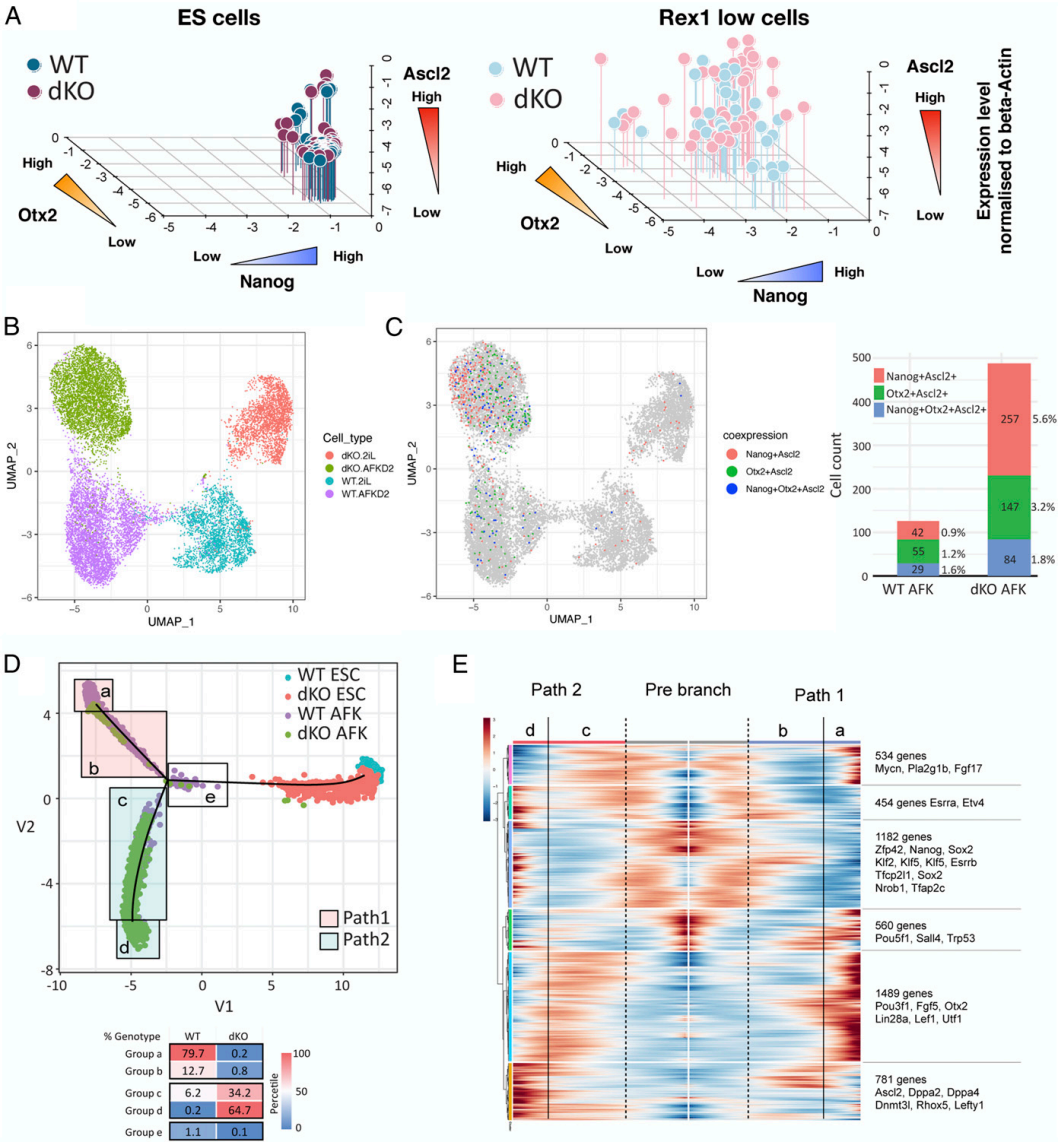
*Dnmt3a/b*-deficient ES cells adopt a deviant fate trajectory to postimplantation trophoblast. (*A*) Single-cell qRT-PCR analysis of ES cells and sorted Rex1::GFP^Lo^ AFK cells. Expression levels of Nanog (*x* axis), Otx2 (*y* axis), and Ascl2 (*z* axis) were normalized to beta-actin. (*B*) UMAP of parental and dKO ES cells and AFK 48-h cells. (*C*) UMAP colored to show cells coexpressing Nanog^+^Ascl2^+^ (red), Otx2^+^Ascl2^+^ (green, or Nanog^+^Otx2^+^Ascl2^+^ (blue) (*Left*) with enumeration (*Right*). (*D*) Pseudotime ordering. Proportions of each genotype of AFK 48-h cells in areas *a*– *e* are shown below. (*E*) Gene expression heatmap of 5,000 differentially expressed genes in pseudotime.

We examined the initial misregulation of gene expression by single-cell qRT-PCR. We used *Nanog* (naive), *Otx2* (formative), and *Ascl2* (trophoblast) as representative markers (Fig. 2*A* and *SI Appendix*, Fig. S2*A*). The majority of parental cells down-regulated *Nanog* and gained *Otx2* after 48 h in AFK. Dnmt3dKO cells similarly gained Otx2 but generally retained higher Nanog levels. Most strikingly, *Ascl2* was up-regulated in more than half of the Dnmt3dKO cells and many of these were also positive for both Nanog and Otx2. Unexpectedly, we also detected a fraction of triple positive cells among parental cells (Fig. 2*A* and *SI Appendix*, Fig. S2*A*).

We extended this analysis using the 10× Genomics platform for single-cell RNA-sequencing (scRNA-seq). Uniform manifold approximation and projection (UMAP) analysis clustered cells by culture condition in the first dimension and by genotype in the second dimension (Fig. 2*B*). We inspected markers for pluripotency states, germ layers, and trophoblast (*SI Appendix*, Fig. S2*B*). In ES cells, expression was not significantly altered between parental and mutant. However, in mutant 48-h AFK cells we observed persistent expression of multiple naive genes, lower up-regulation of formative genes, and ectopic expression of several trophoblast genes, though not Elf5 or Cdx2, which were detected in only 0.1 and 0.7% of cells, respectively. We examined *Nanog*, *Otx2*, and *Ascl2* using a unique molecular identifier (UMI) count above zero to classify expression. *Nanog*^+^*Ascl2*^+^, *Otx2*^+^*Ascl2*^+^, and *Nanog*^+^*Otx2*^+^*Ascl2*^+^ cells were present in both parental and Dnmt3dKO cells at 48 h (Fig. 2*C*). However, the combined proportion of dual and triple positive cells involving Ascl2 was three times higher in the mutants, consistent with single-cell qRT-PCR.

Pseudotime analysis using Monocle 2 (23) indicated a branchpoint in differentiation trajectory (Fig. 2*D*). We arbitrarily partitioned cells at and after the branchpoint into five groups (*a*–*e*). Parental cells were predominantly distributed along path 1, whereas mutant cells were almost exclusively located on path 2. Notably, however, 6.4% of parental cells initiated path 2, although very few reached the endpoint. Differentially expressed genes in parental cells featured formative markers on path 1 and trophoblast genes on path 2 (*SI Appendix*, Fig. S2*C*). Differential expression analysis without considering genotypes substantiated these alternative fates (Fig. 2*E*).

We investigated relatedness between path 2 and in vivo trophoblast. From published data (24) we identified genes up-regulated in E3.5 trophectoderm or E6.5 trophoblast relative to ICM and postimplantation epiblast, respectively. Correlation was low for E3.5 trophectoderm-enriched genes with either pathway. In contrast, many E6.5 trophoblast-enriched genes were up-regulated on path 2 (*SI Appendix*, Fig. S2*D*).

### Chromatin Accessibility and Ascl2 Misexpression in the Absence of De Novo DNA Methylation.

**Fig. 3.**
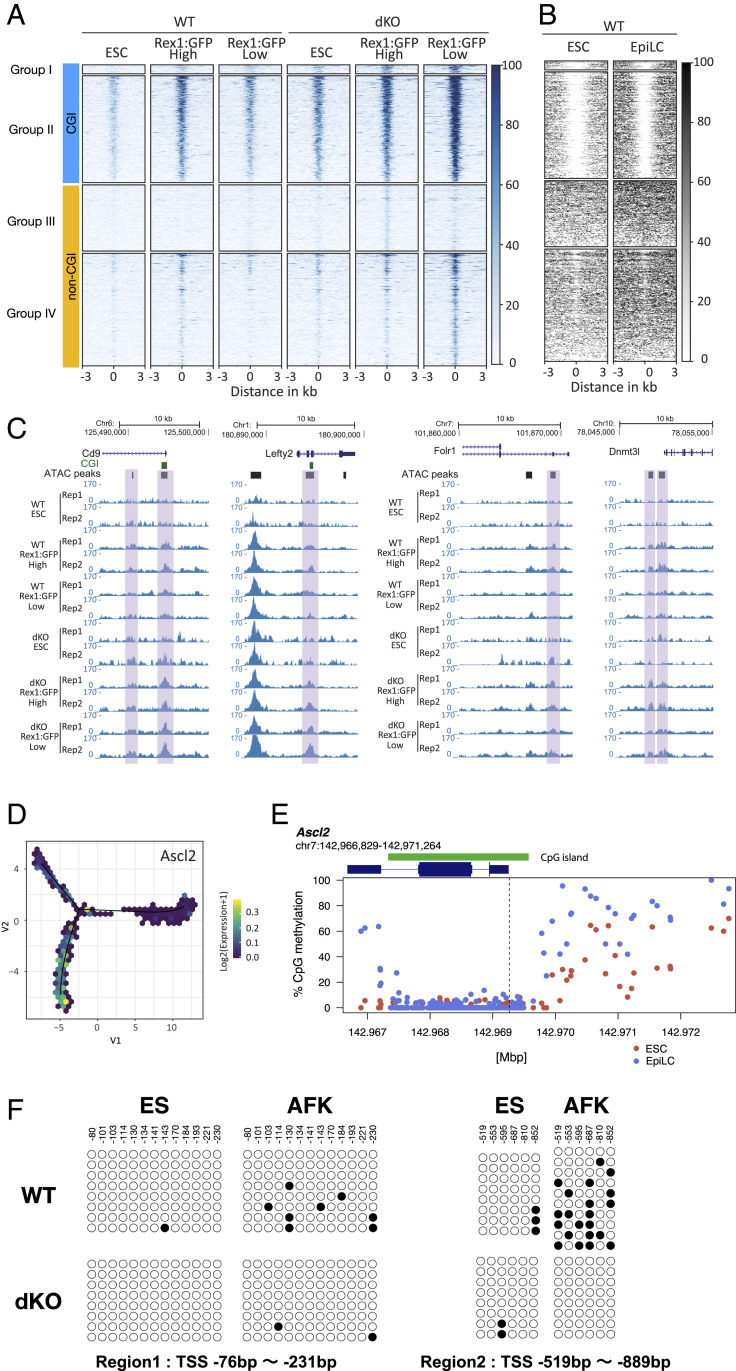
*Ascl2* locus opens during formative transition and remains open in *Dnmt3a/3b* mutants. (*A*) Heatmaps of ATAC-seq intensity distributions grouped according to increased accessibility in Dnmt3dKO GFP^Hi^ (I and III) or GFP^Lo^ (II and IV) presence or absence of CGI. (*B*) Heatmap of CpG methylation across ATAC-seq peaks in ES cells and EpiLCs (10). (*C*) Genome browser examples of genes that are more open in Dnmt3dKO cells. (*D*) Expression of *Ascl2* in pseudotime. (*E*) *Ascl2* gene locus methylation pattern during ES-to-EpiLC transition (data from ref. 10). Dashed line marks the TSS. (*F*) Bisulfite Sanger sequence analysis of *Ascl2* TSS upstream sequence. Region 1 is within CGI and region 2 is within the CGI shore. Filled circles represent methylated cytosine and open circles represent unmethylated cytosine. At least eight clones each were sequenced.

Chromatin is remodelled during formative transition (25). We used assay for transposase accessible chromatin coupled to deep sequencing (ATAC-seq) (26) to survey chromatin accessibility in the absence of de novo DNA methylation. We analyzed ES cells, 48-h GFP^Hi^ transitional cells, and GFP^Lo^ posttransition cells and identified loci that are more open at 48 h in Dnmt3dKO cells than parental cells (Log2 fold change >0.7, *P* value <0.05). We classified four groups according to opening in transitional (GFP^Hi^, groups I and III) or posttransition populations (GFP^Lo^, groups II and IV), and presence or absence of a CpG island (CGI), annotated by the University of California Santa Cruz (UCSC) genome browser (Fig. 3*A*). The strongest peaks were associated with CGIs, which were lowly methylated at all stages (Fig. 3*B* and *SI Appendix*, Fig. S3 *A* and *B*). Non-CGI peaks were weaker and within regions that are methylated in parental cells, although a short stretch of reduced methylation was apparent at many group IV peaks (Fig. 3*B* and *SI Appendix*, Fig. S3 *A* and *B*). As expected, CGI peaks are mainly associated with annotated promoters and non-CGI peaks with enhancers (*SI Appendix*, Fig. S3*C*).

The analysis revealed chromatin regions that opened transiently in parental cells but remained accessible in Dnmt3dKO cells (groups II and IV, Fig. 3*A* and *SI Appendix*, Fig. S3*B*). Example genome browser tracks are shown in Fig. 3*C*. Genes associated with these peaks showed high correlation with differentially expressed genes in Dnmt3dKO GFP^Lo^ cells (*SI Appendix*, Fig. S3*D*). We also found a positive correlation with the set of E6.5 trophoblast-enriched genes (*SI Appendix*, Fig. S3*E*).

Thus, during exit from naive pluripotency, Dnmt3dKO ES cells fail to close down loci that normally open transiently during transition. These regions encompass promoters and proximal enhancers for a subset of E6.5 trophoblast genes that become misexpressed.

Transcription factor motif enrichment analysis across ATAC peaks at CGI loci in Dnmt3dKO GFP^Lo^ cells identified, among others, Ascl1 (*SI Appendix*, Fig. S3*F*). Ascl1 was not expressed in any of the samples studied, but the motif is shared with Ascl2, which, as noted above, is rapidly up-regulated in transitioning mutant cells (Fig. 3*D*). The Ascl motif was present in 531 of 609 promoter regions (−2,000 to +500 bp around the transcription start site [TSS]) of E6.5 trophoblast-enriched genes. In parental ES cells the *Ascl2* locus opened during formative transition (*SI Appendix*, Fig. S3*G*) but in Dnmt3dKO cells, *Ascl2* promoter accessibility increased further posttransition (*SI Appendix*, Fig. S3*G*).

Inspection of published bisulfite sequencing data (10) revealed as expected that the *Ascl2* CGI is not methylated in ESCs or during naive-to-EpiLC transition. However, the flanking CGI shore gained methylation during transition (Fig. 3*E*). We confirmed gain of CGI shore methylation in parental cells in AFK that did not occur in dKO cells (Fig. 3*F*). CGI shores are thought to contribute to regulation of CGI genes (27). The CGI shore sequence (1,807 bp, within 2 kb upstream of the TSS) contains five Ascl2 motifs identified by JASPAR (28), consistent with autoactivation potential as reported in intestinal stem cells (29).

Previously it was found that ES cells deficient for Dnmt3a/3b gradually lost DNA methylation and after multiple passages could no longer form teratomas (14). That study was performed on ES cells cultured in serum, which have a hypermethylated genome compared to the early embryo. In 2iL medium used here the genome is hypomethylated, similar to the embryo (6–8). To examine the acute effect of *Dnmt3a/3b* depletion we used selection for integration of gRNA and Cas9 expression vectors to achieve rapid enrichment for targeted cells. We transfected two ES cell lines and after 3 d, cells were transferred to AFK for a further 48 h. qRT-PCR with primers spanning the deletion registered a reduction in *Dnmt3a* and *Dnmt3b* transcripts indicating effective genome editing. The actual mutation frequency is likely to be higher due to small indels which are not detected. In the mixed populations, assayed almost immediately after *Dnmt3a/3b* depletion, we detected up-regulation of *Rhox6*, a gene previously reported to be regulated by DNA methylation (30), together with *Ascl2* (*SI Appendix*, Fig. S3*H*). We carried out bisulfite sequencing of the *Ascl2* locus following *Dnmt3a/3b* deletion as above. We detected gain of methylation in the CGI shore in parental cells after 48 h in AFK, that was reduced in the *Dnmt3a/3b* mutant population (*SI Appendix*, Fig. S3*I*). These findings indicate a direct effect of *Dnmt3a/3b* depletion on both expression and methylation of *Ascl2*.

Imprinted silencing of the paternal allele of *Ascl2* in placenta does not involve DNA methylation (31). Instead, the imprinted lncRNA *Kcnq1ot1* suppresses transcription in *cis* (32). We inspected levels of *Kcnq1ot1* and found no change in Dnmt3dKO cells, indicating that loss of imprinting is not responsible for up-regulation of *Ascl2* (*SI Appendix*, Fig. S3*J*).

**Fig. 4.**
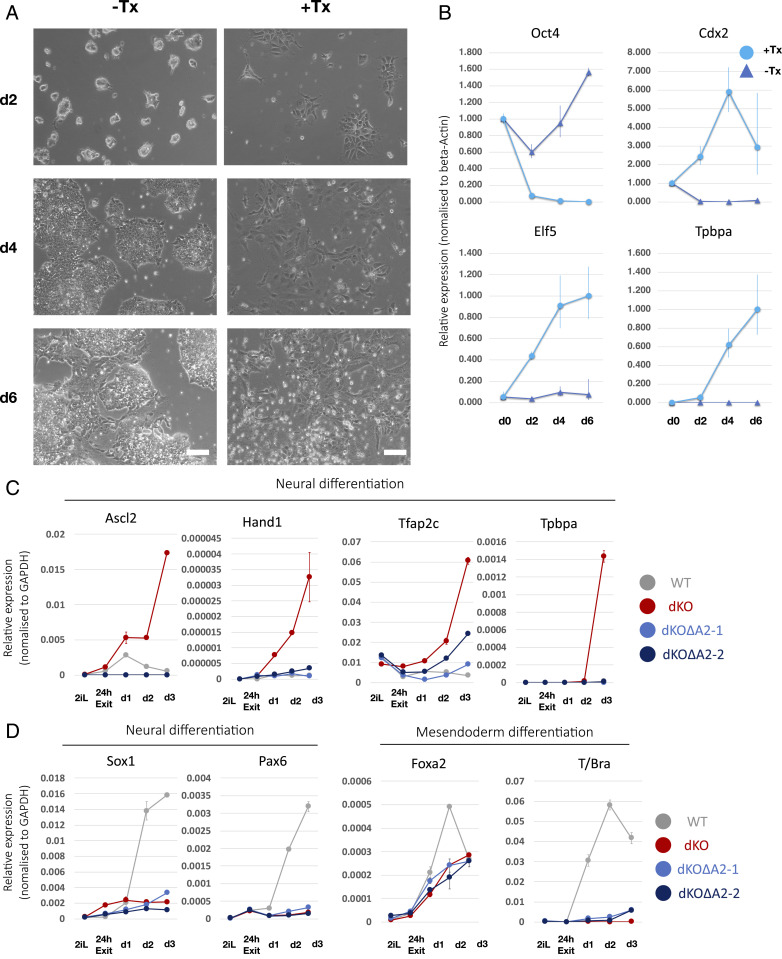
Ascl2 propels transdifferentiation to trophoblast-like cells. (*A*) Images of ES cells cultured in serum/LIF with or without Tx induced activation of Ascl2–ER. (Scale bars, 100 µm.) (*B*) qRT-PCR analysis of marker expression during Ascl2–ER induction. (*C*) qRT-PCR analysis of trophoblast marker expression in neural differentiation culture by two clonal lines of Dnmt3dKO∆A2 cells. (*D*) Analysis of somatic lineage marker expression by Dnmt3dKO∆A2 cells in neural and mesendoderm induction protocol. Error bars represent SD from technical triplicates (*B*) or duplicates (*C* and *D*).

We then investigated whether expression of *Ascl2* is sufficient to impose trophoblast-like differentiation. For this we introduced an Ascl2–estrogen receptor (ER) fusion construct into parental ES cells. Upon tamoxifen (Tx) treatment in serum and LIF, cells changed morphology within 2 d, becoming larger and flattened (Fig. 4*A*). *Oct4* was down-regulated and trophectoderm genes *Elf5*, *Cdx2*, and *Tpbpa* were up-regulated (Fig. 4*B*). Thus, misexpression of *Ascl2* can initiate trophoblast-like differentiation.

Finally, we tested whether activation of *Ascl2* was necessary for trophoblast-like differentiation of Dnmt3dKO cells. We deleted *Ascl2* to create Dnmt3dKOΔA2 cells (*SI Appendix*, Fig. S4*A*) and saw that the misregulation of trophoblast genes was abolished (Fig. 4*C*). Furthermore, at 24 h, Dnmt3dKOΔA2 cells expressed *Otx2*, *Oct6*, and *Fgf5* formative markers (*SI Appendix*, Fig. S4*B*). However, neural genes were not subsequently up-regulated and mesendodermal differentiation remained inefficient (Fig. 4*D*), indicating later differentiation defects unrelated to Ascl2.

### Dnmt3a/b Deficient Cells Are Outcompeted by Wild-Type Cells.

**Fig. 5.**
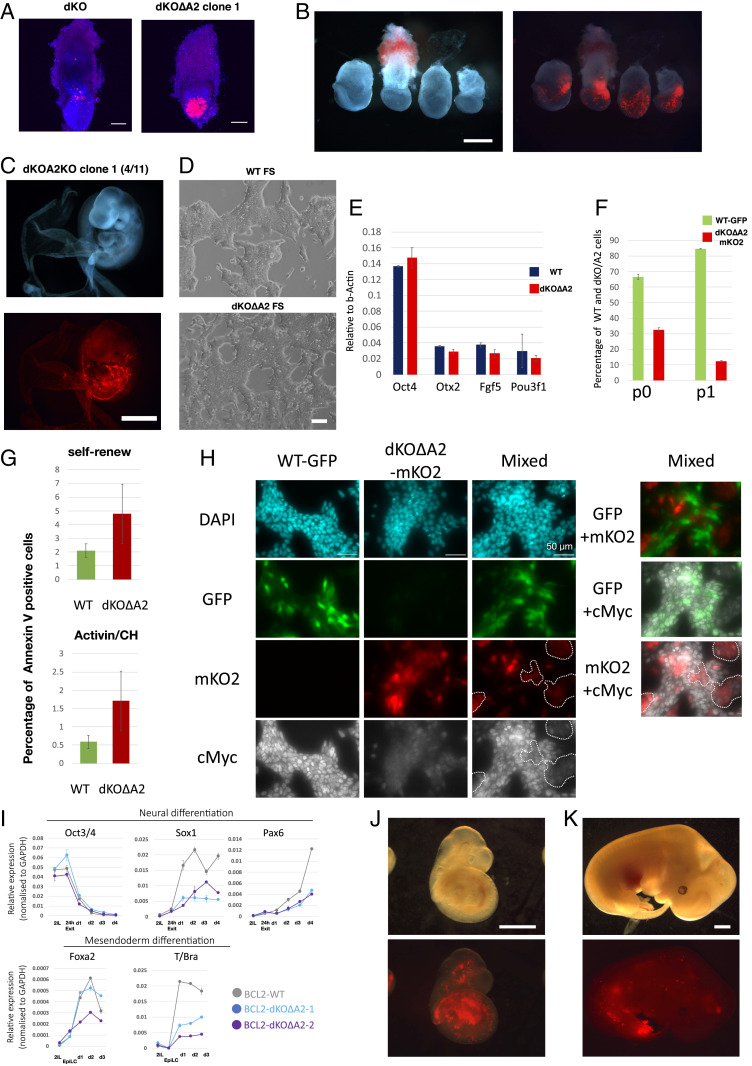
*Dnmt3a/b*-deficient cells are outcompeted by wild-type cells and do not persist in chimeras. (*A*) Maximum projection confocal images of chimeric contributions to E6.5 embryos. Red, mKO2; blue, Eomes immunostaining. (Scale bar, 100 μm.) (*B*) Dnmt3dKO∆A2 ES cell chimeras at E7.5. The 25% opacity brightfield images are overlaid onto fluorescent image. (Scale bar, 250 µm.) (*C*) Dnmt3dKO∆A2 ES cell chimeras at E9.5. (Scale bar, 1 mm.) (*D*) FS cells established from parental and Dnmt3dKO∆A2 ES cells. (Scale bar, 100 µm.) (*E*) qRT-PCR analysis of FS cell markers. Error bar represents SD from technical duplicates. (*F*) Percentage of GFP-labeled WT FS cells and mKO2-labeled dKO-A2 FS cells in the mixed coculture. Passage zero (p0) is 2 d after plating. Error bar represents SD from two cultures. (G) Annexin V positive cells quantified by flow cytometry after 24 h in indicated coculture. Error bars represents SD from six experiments. *P* < 0.05. (*H*) Immunofluorescence images of cMyc parental FS cells (GFP), Dnmt3dKO∆A2 FS cells (mKO2), and coculture for 24 h. Dashed regions highlight mKO2 positive cells in coculture. (*I*) qRT-PCR analysis of marker expression during neural and mesendoderm differentiation of BCL2-transfected ES cells. Error bar represents SD from technical duplicates. (*J*) BCL2-Dnmt3dKO∆A2 ES cell chimeras at E9.5. The 20% opacity brightfield images are overlaid onto fluorescent images. (Scale bar, 1 mm.) (*K*) BCL2-Dnmt3dKO∆A2 ES cell chimeras at E12.5. (Scale bar, 1 mm.)

We investigated whether deletion of *Ascl2* may restore contribution to chimeric epiblasts. We saw substantial colonization by Dnmt3dKOΔA2 cells in 5 of 10 epiblasts at E6.5 (Fig. 5*A* and *SI Appendix*, Fig. S5*A*). Moreover, we observed no donor cells in extraembryonic regions. At E7.5, contributions were reduced and appeared biased to the posterior region (Fig. 5*B* and *SI Appendix*, Fig. S5*B*). Immunostaining for Stella and Tfap2c showed no enrichment for primordial germ cells (PGCs) (*SI Appendix*, Fig. S5 *C* and *D*). By E9.5, we observed only sparse contributions in 4 of 11 embryos (Fig. 5*C* and *SI Appendix*, Fig. S5*E*).

Progressive dilution of mutant cells in chimeras suggested a competitive disadvantage with wild-type host epiblast cells (33). To examine this possibility, we used formative pluripotent stem (FS) cells related to E6.0 epiblasts (34). We were unable to establish FS cells from Dnmt3dKO cells, likely due to disruptive effects of *Ascl2* misexpression. However, Dnmt3dKOΔA2 ES cells could be converted into stable FS cell lines that expressed the core pluripotency factor Oct4 together with formative markers (Fig. 5 *D* and *E*). We cocultured equal numbers of GFP expressing wild-type and mKO2 expressing Dnmt3dKOΔA2 FS cells. Proportions of each genotype were quantified by flow cytometry. We observed reduction in the fraction of mutant cells by passage 0 that was further increased by passage 1 (Fig. 5*F*). In cocultures Annexin V staining was higher for Dnmt3dKO∆A2 FS cells during self-renewal and mesendoderm induction (*P* < 0.05) though not neural induction (*P* > 0.05) (Fig. 5*G* and *SI Appendix*, Fig. S5*G*).

Relative level of cMyc is a key determinant in cell competition (35–37). By immunostaining we saw that Dnmt3dKOΔA2 FS cells have lower levels of cMyc protein than wild-type cells (Fig. 5*H*). Immunoblotting confirmed that cMyc expression was reduced in mutant cells (*SI Appendix*, Fig. S5*F*). These observations suggest that cMyc-based cell competition may cause elimination of Dnmt3dKO cells mixed with wild-type cells (36) and that inactivation of *Ascl2* avoids only the first round of competition. The mechanism that reduces cMyc is unclear, but we surmise is secondary to other perturbations in the absence of de novo methylation.

We introduced *BCL2* into Dnmt3dKOΔA2 ES cells. Under in vitro differentiation conditions *BCL2* transfectants showed up-regulation of *Sox1*, *Pax6*, *T*, and *Foxa2*, although still below the levels observed for parental transfectants (Fig. 5*I*). After blastocyst injection we recovered five chimeras among seven embryos at E9.5. Contributions were variable, but substantial in three of the five (Fig. 5*J*). We also collected embryos at E12.5 and detected mKO2 fluorescence in two of the four specimens, with donor-derived cells in neural tissues and mesenchyme (Fig. 5*K* and *SI Appendix*, Fig. S5*H*). These contributions were noticeably lower than at E9.5, however, suggesting ongoing loss of *Dnmt3a/b*-deficient cells even in the presence of a strong survival factor.

## Discussion

Our findings indicate that de novo DNA methylation safeguards the formative pluripotency transition to somatic lineage competence. In the absence of Dnmt3a and Dnmt3b, cells exiting naive pluripotency are liable to adopt a deviant fate trajectory and develop features of postimplantation trophoblast. Notably, a small fraction of parental ES cells initiates the trophoblast transdifferentiation trajectory but they do not continue on this path because de novo DNA methylation prevents full activation of the trophoblast program. Our study identifies Ascl2 as a trophoblast determination gene that is potentially directly regulated by de novo DNA methylation. Misexpression of Ascl2 can provoke ES cell transdifferentiation. Conversely, removal of *Ascl2* eliminates the aberrant fate trajectory in *Dnmt3a/3b* mutant cells and restores pluripotency progression. *Dnmt3a/3b*-deficient cells remain compromised in later differentiation, however, and are unable to compete with wild-type cells in the chimera context.

Deletion of *Dnmt3a/3b* in ES cells generated in serum and LIF culture was previously shown to result in progressive erosion of methylation at repetitive sequences and, after long-term culture, a failure to produce teratomas (14). Effects of continuous culture on transcription or lineage commitment were not characterized. ES cells in serum are subject to heterogeneous and dynamic hypermethylation (38) whereas in 2i/LIF the genome is hypomethylated, similar to the inner cell mass (7, 39). The requirement for de novo methylation may be more acutely apparent in ground state ES cells in 2i/LIF due to the lower basal level of methylation. Crucially, the phenotype of *Ascl2* misregulation and trophoblast-like differentiation is specifically associated with absence of the de novo Dnmts and is eliminated by their restoration. The CGI shore adjacent to the *Ascl2* promoter is subject to de novo DNA methylation during formative transition, suggestive of a potential direct silencing effect. Alternatively, an upstream activator of *Ascl2* may be silenced by de novo methylation.

Regardless of the mechanism, our findings are consistent with detection of Ascl2 misexpression in *Dnmt3a/3b* dKO embryos at E9.5 (30). Inspection of recent transcriptome data from *Dnmt3a/3b*-deficient embryos at E8.5 (40) also revealed low but significant (*P* < 0.01) up-regulation of *Ascl2*. Nonetheless, ectopic trophoblast differentiation is not the major cause of embryonic lethality. Heightened susceptibility of ES cells to this fate alteration may reflect adaptation to the in vitro environment or the absence of constraints operative in the embryo. It is also important to note that deregulation of *Ascl2* does not instruct normal trophoblast lineage development but triggers a deviant differentiation process and generation of an aberrant cell phenotype. Our findings serve as a cautionary note for interpretation of lineage potential and differentiation from pluripotent stem cells, in particular claims of expanded potency after exposure to epigenome modifying agents or other perturbations.

Overall, our results demonstrate that in the absence of de novo DNMTs the ability to execute a cell state transition is compromised due to misexpression of a gene with fate switching potency. The example of *Ascl2* illustrates how genome-wide methylation may have been coopted to constrain expression of a pivotal gene that is transiently accessible during chromatin reconfiguration. This scenario may replay at other critical loci that open incidentally in the course of cell transitions. Indeed, transcriptome analysis of *Dnmt3a/3b* dKO embryos has highlighted deregulated expression of lineage-specific genes at E8.5 (40). The multiple abnormalities in *Dnmt3a/3b* mutant embryos and the inability of mutant ES cells to persist in any lineage (39) might be explained by a recurrent requirement for DNA methylation at critical loci to safeguard transcriptome trajectories. In this context it is of interest that mutations in *DNMT3A* and *DNMT3B* are associated with tumorigenesis in various tissues (41, 42), possibly relating to corruption of cellular transitions.

## Materials and Methods

### Cell Culture.

ES cells were maintained in 2iL medium on gelatin-coated plates as described (18). 2iL medium consists of 10 ng/mL of mouse LIF, 1 µM of Mek inhibitor PD0325901, and 3 µM of GSK3 inhibitor CHIR99021 in N2B27 basal medium. EpiLCs were induced by plating 2 × 10^5^ ES cells in 20 ng/mL Activin A, 12.5 ng/mL bFgf, and 1% knockout serum replacement (KSR), in N2B27 medium on fibronectin-coated six-well plates. GFP high or low fractions were collected using MoFlo (Beckman Coulter) or FACS Fusion (BD) instruments. Neural differentiation was induced by plating 1 × 10^5^ cells in N2B27 basal medium on laminin-coated six-well plates. Mesendoderm induction was performed by plating 1 × 10^5^ cells in 10 ng/mL Activin A and 3 μM CHIR99021 in N2B27 medium on fibronectin-coated six-well plates. Trophoblast culture media were formulated as described previously (19, 20). For chimera studies, cells were stably transfected with pPBCAG-mKO2-IP plasmid by TransIT LT1 (Mirus) with pCAG-PBase plasmid and selected with 1 µg/mL puromycin. Human *BCL2* was introduced by pT2PyCAG-hBCL2-IH by TransIT LT1 with pCAG-T2ase plasmid (43) and selection with 100 µg/mL of hygromycin B. To establish tamoxifen-inducible *Ascl2* expression lines, pPBCAG-Ascl2ER-IN plasmid was cotransfected with pCAG-PBase plasmid into E14Tg2a ES cells and clones picked after selection with 250 µg/mL G418. DnmtdKO∆A2 FS cells were established by conversion of ES cells and maintained in 3 ng/mL of Activin A, 2 µM XAV939, and 1 µM BMS493 in N2B27 medium on fibronectin-coated plates as described (34). Alexa Fluor 647 conjugated Annexin V (BioLegend, Cat. No. 640911) was used for apoptotic cell detection by FACS Fortessa (BD).

### Gene Targeting.

*Dnmt3a* or *Dnmt3b* single KO and double KO cells were established previously using CRISPR/Cas9 and gRNAs designed to excise sequences encoding the catalytic domain (15). Knockout cells were used within 15 passages and compared with wild-type cells of similar passage. To assess the effect of gene knockout acutely, gRNAs were cloned into a piggyBac vector with puromycin resistance cassette, pCML32 (34), and cotransfected with pPB-Cas9-IN and pCAG-PBase using TransIT-LT1 (Mirus) followed by selection with 1 µg/mL puromycin and 300 µg/mL G418. To rescue Dnmt KO cells, pPB-CAG-Dnmt3a-IP and pPBCAG-Dnmt3b-IZ were cotransfected with pCAG-PBase using TransIT-LT1 with selection in 1 µg/mL puromycin and 40 µg/mL zeocin. *Ascl2* gRNAs were designed to excise exon2, which encodes the full-length protein. Clones were screened by genomic PCR. gRNAs and primers are listed in *SI Appendix*, Table S1.

### Chimeric Embryo Experiments.

Reporter-expressing ES cells were dissociated and 10 to 15 cells injected into each blastocyst. Injected embryos were transferred to the uteri of pseudopregnant females or cultured in vitro for 24 h in M2 medium (Sigma-Aldrich) in 7% CO_2_ at 37 °C.

### Immunostaining.

Embryos and culture cells were fixed with 4% paraformaldehyde (PFA) at room temperature (for 15 min for cells and up to E7.5 stage embryos, 60 min for E9.5 and E12.5). Whole E6.5 embryos were stained with rabbit anti-Eomes antibody (Abcam, Cat. No. ab23345). Blastocysts were stained with rabbit anti-GFP (Invitrogen, Cat. No. A-11122) and rat anti-Sox2 (eBioscience, Cat. No. 14-9811-82). After the fixation, E9.5 and E12.5 embryos were incubated with 20% sucrose/phosphate buffered saline (PBS) overnight at 4 °C, then embedded in OCT cryo-embedding compound (Sakura, Cat. No. 4583). Sections were stained with rat anti-Sox2 (eBioscience) and fluorescein isothiocyanate (FITC)-conjugated mouse anti-cTnT antibodies (Abcam, Cat. No. ab105439) (E9.5 only). Nuclei were stained with DAPI. Embryos and sections were imaged by either a Leica SP5 or Zeiss LSM880 confocal microscope. E7.5 PGC were stained with anti-Tfap2c (Santa Cruz, Cat. No. sc-8977), anti-Stella (Abcam, Cat. No. ab19878), and rat anti-Sox2 (eBioscience, Cat. No. 14-9811-82). FS cells were stained with rabbit anti-cMyc antibody (Abcam, Cat. No. ab30272).

### Western Blot.

Cells were lysed with RIPA buffer in the presence of protease/phosphatase inhibitor mixture (Invitrogen). Lysed cells were rotated for 20 min and sonicated in a Bioruptor (Diagenode). Cell lysates were cleared by centrifugation, and the supernatant was recovered. Protein concentrations were measured by the bicinchoninic acid (BCA) method (Pierce). A total of 15 µg of protein was loaded in each well. Blots were blocked with 5% BSA/TBS 0.1% Triton-X for 1 h at room temperature and incubated overnight with primary antibodies at 4 °C. Secondary antibodies were incubated for 1 h at room temperature and signals were detected with ECL Select (GE Healthcare) and Odyssey Fc (Li-Cor). NaOH (0.2 N) was used for stripping. Anti-cMyc (Abcam, Cat. No. ab30272) and anti–α-tubulin (Thermo Fisher Scientific, A-11126) were used.

### qRT-PCR.

Total RNA was isolated with Relila RNA miniprep systems (Promega) and cDNA was synthesized using the GoScript Reverse Transcriptase system (Promega). qRT-PCR was performed with Taqman Gene Expression (Thermo Fisher Scientific) or Fast SYBR Green Master Mix (Thermo Fisher Scientific). Primers are listed in *SI Appendix*, Table S1.

### Bisulfite Sanger Sequencing Analysis.

Genomic DNA was prepared with the DNeasy Blood and Tissue Kit (Qiagen). Purified genomic DNA was treated with an Imprint DNA modification kit to perform bisulfite conversion. Ascl2 promoter regions were amplified by nested PCR with the Touchdown protocol with LongAmp DNA Taq polymerase (New England Biolabs). The PCRs for both rounds were performed as follows: denaturing at 94 °C for 30 s, 10 cycles of gradient PCR, 94 °C for 15 s, 65 °C (annealing temperature reduced 1 °C per cycle) for 15 s, 65 °C for 30 s, and 35 cycle of 94 °C for 15 s, 56 °C for 15 s, and 65 °C for 30 s. A total of 2 µL of first PCR product was used for the nested PCR. PCR products were purified from agarose gel and cloned into TOPO cloning vector (Thermo Fisher Scientific). The sequence results were analyzed with QUMA (44).The primers are listed in *SI Appendix*, Table S1.

### Single-Cell qPCR.

Custom Taqman probes were ordered for Nanog, Otx2, Ascl2, and beta-actin (Thermo Fisher Scientific). Single cells were collected directly into 96-well plates by flow cytometry (MoFlo, Beckman Coulter) and reverse transcription was performed as described previously (45). Cells that had Ct value of beta-actin >14 and no amplification were excluded from the analysis.

### Single-Cell RNA-Seq.

ES cells and 48-h AFK cells were dissociated with accutase (BioLegend) and live cells were collected by flow cytometry (MoFlo, Beckman Coulter). Single-cell cDNA libraries were constructed using 10× Genomics technology and sequenced by the Genomics Core Facility, Cancer Research UK Cambridge Institute.

### ATAC-Seq.

A total of 50,000 ES cells and AFK cells at 48 h were collected by flow cytometry (MoFlo, Beckman Coulter). Cells were washed with ice-cold PBS once then lysed in the buffer (10 mM Tris⋅HCl, pH 7.4, 10 mM NaCl, 3 mM MgCl_2_ 0.1% IGEPAL CA-630). The nuclear pellets were collected and Tn5 tagmentation and library construction were performed with the Illumina Nextera kit (FC-121-1030). DNA was purified with AMPure XP beads (Beckman Coulter).

### Data Analysis.

#### Single-cell RNA-seq analysis.

Preliminary sequencing results (bcl files) were converted to fastq files with CellRanger (version 3.0) following the standard 10× Genomics protocol. Barcodes and UMI ends were trimmed to 26 bp, and mRNA ends to 98 bp. Reads were then aligned to the mouse reference genome (mm10) and gene counts were obtained using the GRCm38.92 annotation in CellRanger. We used Seurat (version 3.1.5) (46) to further process the single-cell RNA-seq data. Cells that have between 200 and 5,500 unique gene counts, and with less than 5% mitochondrial gene content were filtered. We analyzed 2,724 parental and 2,459 Dnmt3dKO ES cells with 4,776 parental and 4,563 Dnmt3dKO 48-h cells. Log normalization using a scale factor of 10,000 was performed, and the data were scaled to produce standardized expression values for each gene across all cells (z-score transformation), while also regressing out unwanted variation in the percent of mitochondrial gene content. We determined the top 2,000 most highly variable genes and performed principal component analysis. Visualization was performed using the UMAP projection method on the first 18 principal components. Differential expression was performed using the Wilcoxon rank sum test, using a threshold of Log2 fold change greater than 0.1 and *P* value less than 0.05. The enrichment of TE (E3.5) and ExE (E6.5) genes was visualized using the AddModuleScore function of Seurat to show the average expression of sets of genes. Coexpression of Nanog, Ascl2, and Otx2 was assigned to cells having at least one unique read for each of the genes.

#### Pseudotemporal analysis.

Pseudotime trajectory was calculated using Monocle2 (version 2.14) (47–49) using the method “DDRTree” with the top 1,000 differentially expressed genes between each of the clusters found from the Seurat clustering. To find differences between cells at the end and middle of each branch, the trajectory was split into sections using an arbitrary pseudotime cutoff of 21.4 for path 1 and 22.25 for path 2, based on visualizing the trajectory. To find genes that are differentially expressed along pseudotime the monocle function differentialGeneTest was used with default parameters.

#### Bulk RNA-seq analysis.

Genes enriched in trophoblast over epiblast at E6.5 or in trophectoderm over ICM at E3.5 were determined by differential expression analysis using publicly available datasets (GSE84236). This was done using tximport (50) to load the dataset into DESeq2 (50) for differential expression analysis using a threshold of Log2 fold change >2 and an adjusted *P* value of <0.05.

#### ATAC-seq analysis.

Raw reads were preprocessed and quality filtered using Trim Galore! (https://github.com/FelixKrueger/TrimGalore), and reads shorter than 15 nt were discarded. Processed reads were then aligned to the mouse reference genome (mm10) using bowtie with parameters “-m1 -v1 –best –strata -X 2000 –trim3 1.” Duplicates were removed using Picard tools. Read pairs larger than one nucleosome length (146 bp) were discarded, and an offset of 4 nt was introduced. Peaks were called with MACS2 and parameters “–nomodel –shift -55 –extsize 110 –broad -g mm –broad-cutoff 0.1”. Then the R package DiffBind (51) was used to calculate reads across the merged peaks and calculate differential peaks for each cell type utilizing the edgeR method (52, 53). Peaks were also identified as being within a CpG island or not and assigned genes based on a window of 2 kb around each peak. Scatterplots were plotted using the differential ATAC-seq fold changes from edgeR against the RNA-seq fold changes from Seurat. In each of the quadrants of the scatterplot a Fisher’s exact test was used to calculate whether the differentially expressed genes were overrepresented. Motif analysis was performed by using the findMotifsGenome tool in homer (version v4.10) (54) on peaks that overlapped with CpG islands in the KO using the WT peaks as background sites.

## Supplementary Material

Supplementary File

## Data Availability

The 10×Genomics and ATAC-seq data are deposited in Gene Expression Omnibus (accession no. GSE158347).
