# Supplementary material for: Disabling de novo DNA methylation in embryonic stem cells allows an illegitimate fate trajectory
Source: Proc Natl Acad Sci U S A. 2021 Sep 13;118(38):e2109475118. doi: 10.1073/pnas.2109475118 (PMC8463881; doi:10.1073/pnas.2109475118)
Supplement: Supplementary File [file pnas.2109475118.sapp.pdf]

PNAS
www.pnas.org

Supplementary Information for

Disabling *de novo* DNA methylation in embryonic stem cells allows an illegitimate fate trajectory

Masaki Kinoshita[1,4], Meng Amy Li[1,4], Michael Barber[1], William Mansfield[1], Sabine Dietmann[1] and Austin Smith[1,2,3,5]

[1] Wellcome-MRC Cambridge Stem Cell Institute
Jeffrey Cheah Biomedical Centre
University of Cambridge
Cambridge CB2 0AW
United Kingdom
[2] Department of Biochemistry
University of Cambridge
Cambridge CB2 1GA
United Kingdom
[3] Living Systems Institute
University of Exeter
Exeter EX4 4QD
United Kingdom

[4] Equal contribution
[5] Author for correspondence
Email: austin.smith@exeter.ac.uk

This PDF file includes:

Figures S1 to S5
Table S1

Fig.S1

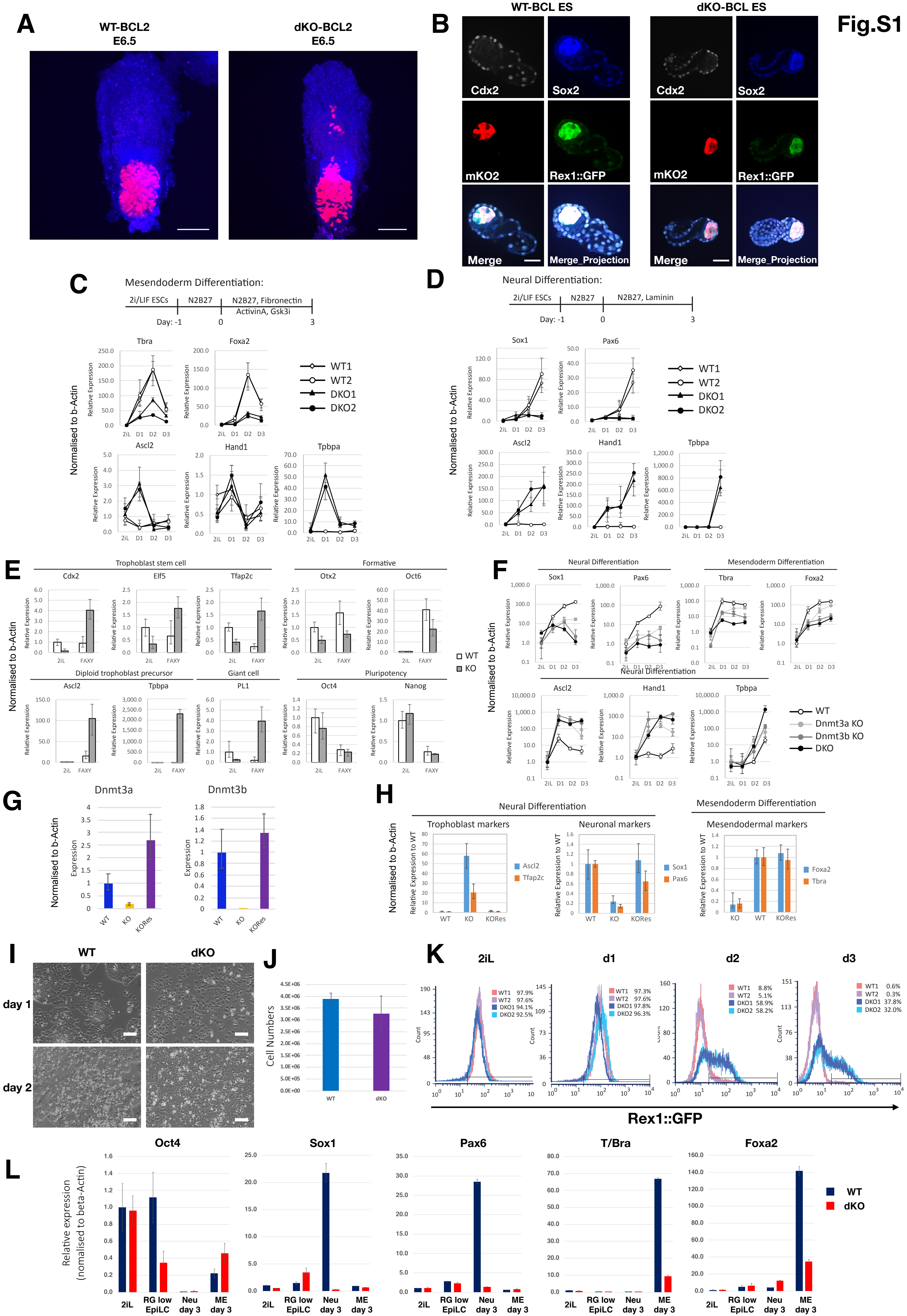

A
WT-BCL2
E6.5
dKO-BCL2
E6.5
B
WT-BCL ES
dKO-BCL ES
Cdx2
Sox2
mKO2
Rex1::GFP
Merge
Merge_Projection
C
Mesendoderm Differentiation:
2i/LIF ESCs
N2B27
N2B27, Fibronectin
ActivinA, Gsk3i
Day: -1
0
3
Normalised to b-Actin
Relative Expression
Tbra
Foxa2
Ascl2
Hand1
Tpbpa
WT1
WT2
DKO1
DKO2
2iL
D1
D2
D3
D
Neural Differentiation:
2i/LIF ESCs
N2B27
N2B27, Laminin
Sox1
Pax6
Ascl2
Hand1
Tpbpa
E
Trophoblast stem cell
Formative
Cdx2
Elf5
Tfap2c
Otx2
Oct6
Diploid trophoblast precursor
Giant cell
Pluripotency
Ascl2
Tpbpa
PL1
Oct4
Nanog
2iL
FAXY
WT
KO
F
Neural Differentiation
Mesendoderm Differentiation
Sox1
Pax6
Tbra
Foxa2
Ascl2
Hand1
Tpbpa
WT
Dnmt3a KO
Dnmt3b KO
DKO
G
Dnmt3a
Dnmt3b
Expression
WT
KO
KORes
H
Neural Differentiation
Mesendoderm Differentiation
Trophoblast markers
Neuronal markers
Mesendodermal markers
Relative Expression to WT
Ascl2
Tfap2c
Sox1
Pax6
Foxa2
Tbra
I
WT
dKO
day 1
day 2
J
Cell Numbers
WT
dKO
K
2iL
d1
d2
d3
Count
WT1 97.9%
WT2 97.6%
DKO1 94.1%
DKO2 92.5%
WT1 97.3%
WT2 97.6%
DKO1 97.8%
DKO2 96.3%
WT1 8.8%
WT2 5.1%
DKO1 58.9%
DKO2 58.2%
WT1 0.6%
WT2 0.3%
DKO1 37.8%
DKO2 32.0%
Rex1::GFP
L
Oct4
Sox1
Pax6
T/Bra
Foxa2
Relative expression
(normalised to beta-Actin)
2iL
RG low EpiLC
Neu day 3
ME day 3
WT
dKO

Fig. S1 (A) Maximum projection confocal microscopy images of BCL2 ES cell chimaeras in Fig 1C, immunostained for Eomes in blue. Scale bar, 100µm. (B) Representative images of blastocysts injected with hBCL2 parental or Dnmt3dKO ES cells and cultured for 24h. Scale bars, 50µm. (C) qRT-PCR analysis of neural lineage and trophoblast markers during neural induction. (D) qRT-PCR analysis of primitive streak and trophoblast markers during mesendoderm induction. (E) qRT-PCR analysis of trophoblast and epiblast markers after seven days in alternative trophoblast cell medium, FAXY (Ohinata and Tsukiyama 2014). (F) RT-qPCR analysis of neural and mesendodrm gene expression in *Dnmt3a* or *Dnmt3b* single mutant ES cells. (G) qRT-PCR assay of *Dnmt3a* and *Dnmt3b* transcripts in WT, dKO and Dnmt3a/b-transfected dKO ES cells using UPL primer pairs spanning the deleted regions. (H) qRT-PCR analysis of somatic lineage and trophoblast marker expression in rescued dKO ES cells. (I) Morphology of WT and dKO cells after 24h and 48h in AFK. Scale bars, 100 µm. (J) Cell counts 48h after plating $2x10^5$ ES cells in AFK. Error bars represent SD from biological replicates (n=3). p>0.05. (K) Rex1::GFP flow cytometry profile during AFK culture for three days. (L) qRT-PCR analysis of 2iL ES cells, Rex1::GFP low 48h AFK cells, and cells differentiated for 3 days post-sorting. All qPCR data are normalized by beta-Actin and error bars represent S.D. from technical duplicates.

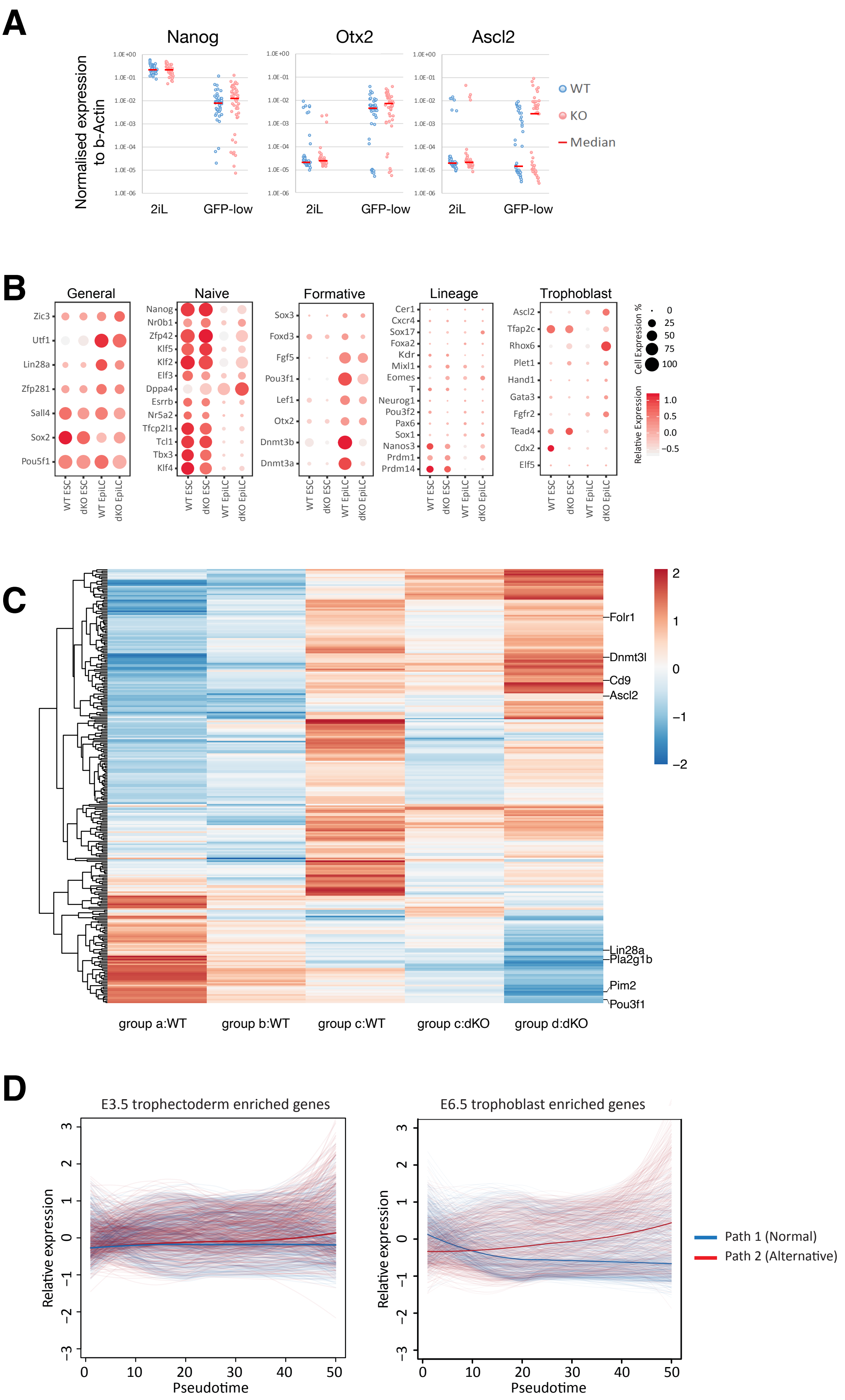
A
Nanog
Otx2
Ascl2
Normalised expression to b-Actin
2iL
GFP-low
WT
KO
Median
B
General
Naive
Formative
Lineage
Trophoblast
Cell Expression %
Relative Expression
WT ESC
dKO ESC
WT EpiLC
dKO EpiLC
C
Folr1
Dnmt3l
Cd9
Ascl2
Lin28a
Pla2g1b
Pim2
Pou3f1
group a:WT
group b:WT
group c:WT
group c:dKO
group d:dKO
D
E3.5 trophectoderm enriched genes
E6.5 trophoblast enriched genes
Relative expression
Pseudotime
Path 1 (Normal)
Path 2 (Alternative)

Fig. S2 (A) Related to Fig. 2A. Single cell qRT-PCR expression levels of Nanog, Otx2 and Ascl2 in undifferentiated ES cells and Rex1-low AFK cells at 48h. (B) Marker gene expression from scRNA-seq data. Size of circles indicates percentage of cells expressing each gene and colour scale indicates expression level. (C) Heatmap of differentially expressed genes (Log2 fold change >0.1 and p-value<0.05) between parental cells in groups b and c from Figure 2d. (D) Expression pattern of E3.5 TE and E6.5 ExE enriched genes along the pseudo-time trajectories, Path 1 in blue and Path 2 in red. Solid lines are mean values. Enriched genes (expression Log2 fold change of 2 and adjusted p-value<0.05) were identified for trophectoderm vs ICM from E3.5 blastocysts and extraembryonic ectoderm vs epiblast from E6.5 embryos (data from Smith Z.D. et al 2017).

Fig.S3

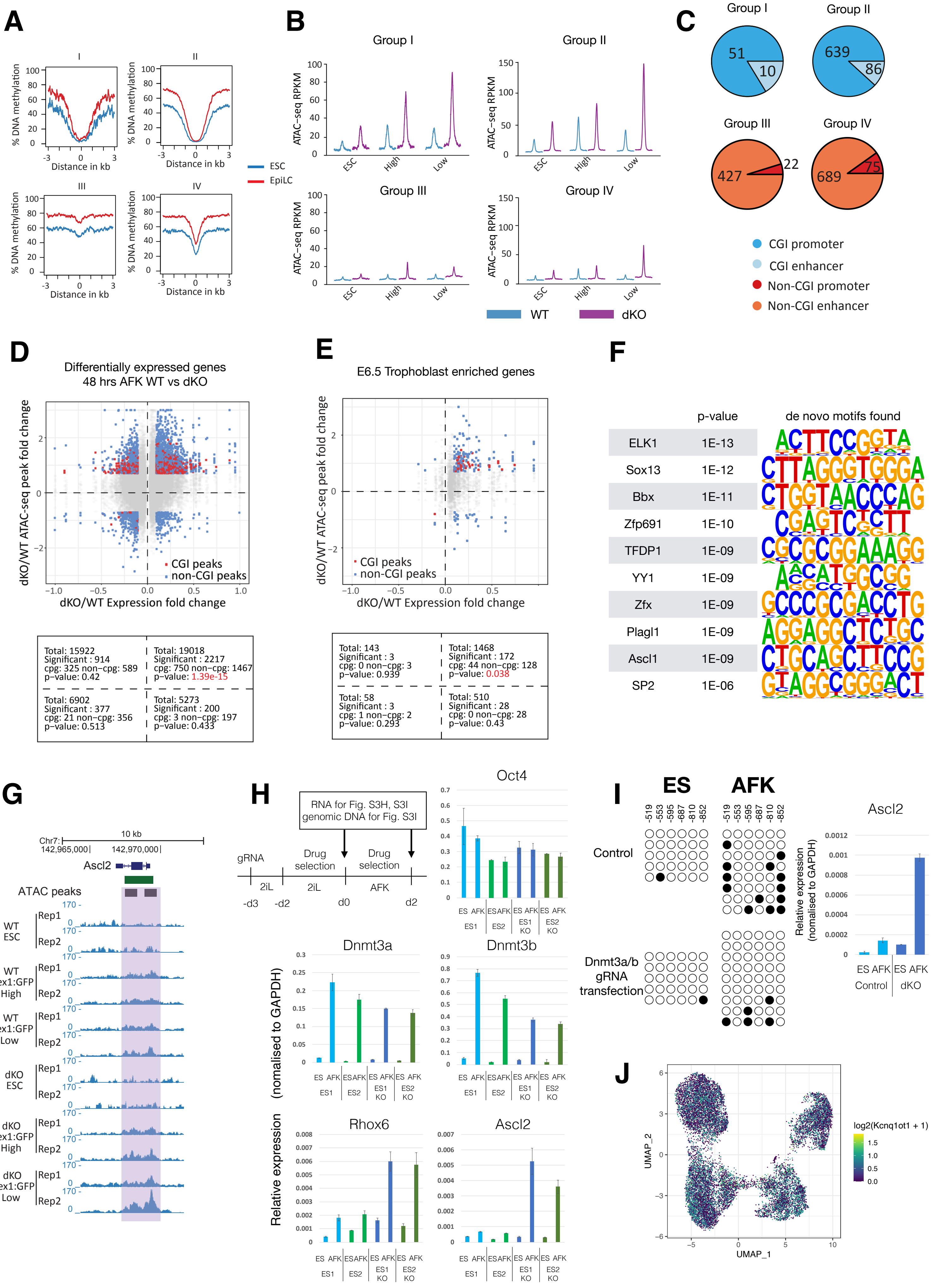
A
% DNA methylation
Distance in kb
ESC
EpiLC
B
Group I
Group II
Group III
Group IV
ATAC−seq RPKM
ESC
High
Low
WT
dKO
C
CGI promoter
CGI enhancer
Non-CGI promoter
Non-CGI enhancer
D
Differentially expressed genes
48 hrs AFK WT vs dKO
dKO/WT ATAC-seq peak fold change
dKO/WT Expression fold change
CGI peaks
non-CGI peaks
Total: 15922
Significant : 914
cpg: 325 non−cpg: 589
p−value: 0.42
Total: 19018
Significant : 2217
cpg: 750 non−cpg: 1467
p−value: 1.39e-15
Total: 6902
Significant : 377
cpg: 21 non−cpg: 356
p−value: 0.513
Total: 5273
Significant : 200
cpg: 3 non−cpg: 197
p−value: 0.433
E
E6.5 Trophoblast enriched genes
Total: 143
Significant : 3
cpg: 0 non−cpg: 3
p−value: 0.939
Total: 1468
Significant : 172
cpg: 44 non−cpg: 128
p−value: 0.038
Total: 58
Significant : 3
cpg: 1 non−cpg: 2
p−value: 0.293
Total: 510
Significant : 28
cpg: 0 non−cpg: 28
p−value: 0.43
F
p-value
de novo motifs found
ELK1 1E-13
Sox13 1E-12
Bbx 1E-11
Zfp691 1E-10
TFDP1 1E-09
YY1 1E-09
Zfx 1E-09
Plagl1 1E-09
Ascl1 1E-09
SP2 1E-06
G
Chr7:
142,965,000
142,970,000
10 kb
Ascl2
ATAC peaks
WT ESC
WT Rex1:GFP High
WT Rex1:GFP Low
dKO ESC
dKO Rex1:GFP High
dKO Rex1:GFP Low
Rep1
Rep2
H
RNA for Fig. S3H, S3I
genomic DNA for Fig. S3I
gRNA
Drug selection
2iL
AFK
-d3
-d2
d0
d2
Oct4
Dnmt3a
Dnmt3b
Rhox6
Ascl2
Relative expression (nomalised to GAPDH)
ES AFK
ES1
ES2
ES1 KO
ES2 KO
I
ES
AFK
Control
Dnmt3a/b gRNA transfection
Relative expression (nomalised to GAPDH)
dKO
J
UMAP_1
UMAP_2
log2(Kcnq1ot1 + 1)

Fig. S3 (A) CpG DNA methylation pattern of identified peaks in WT ES AFK cells in Fig.3A. Averaged percentage of DNA methylation from each group is shown. (B) RPKM values from each group identified in Fig. 3A. (C) Association (within 2kb) of ATAC-seq peaks in groups I-IV promoters or enhancers. (D) Correlation plot for open chromatin regions in GFP low AFK cells with differentially expressed genes between WT and dKO AFK 48h cells. (E) Correlation plot for open chromatin regions with expression in AFK cells of E6.5 trophoblast-enriched genes associated with ATAC peaks. (F) De novo motif analysis of differential ATAC peaks. (G) ATAC-seq profile of the *Ascl2* locus in WT and dKO cells in 2iL and AFK. (H) qRT-PCR analysis in ES cells and AFK day 2 cells immediately after Dnmt3a/3b depletion, as depicted in the schematic. PCR with UPL primer pairs measures Dnmt3a/3b transcript reduction due to the intended deletions but not to all indel events. Results are from two independent ES cell lines. Error bars represent S.D. from technical duplicates. (I) CGI shore CpG methylation analysis after Dnmt3a/3b depletion as above in an independent experiment. Filled circles represent methylated cytosine and open circles unmethylated cytosine. qRT-PCR shows *Ascl2* gene expression level from the same samples. Error bars represent S.D. from technical duplicates. (J) UMAP from Fig 2B coloured to show *Kcnq1ot1* expression values.

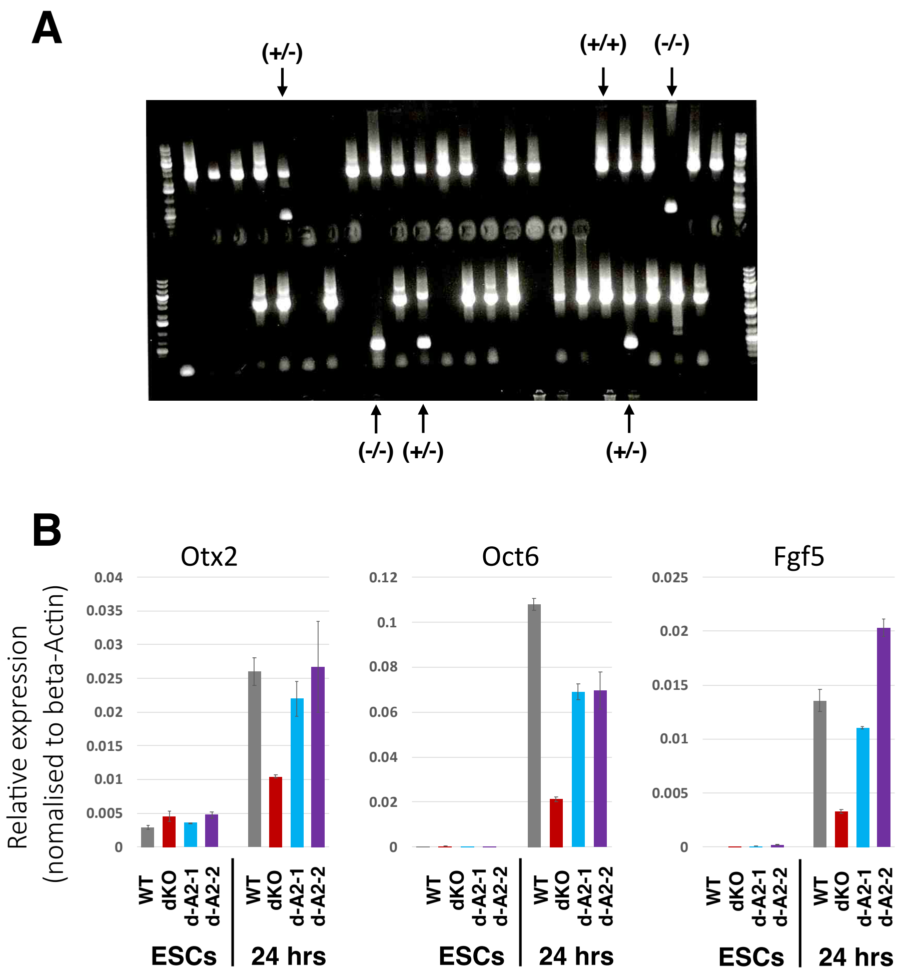
A
(+/-)
(+/+)
(-/-)
(-/-) (+/-)
(+/-)
B
Otx2
Oct6
Fgf5
Relative expression
(nomalised to beta-Actin)
0.04
0.035
0.03
0.025
0.02
0.015
0.01
0.005
0
0.12
0.1
0.08
0.06
0.04
0.02
0
0.025
0.02
0.015
0.01
0.005
0
WT
dKO
d-A2-1
d-A2-2
ESCs
24 hrs

Fig.S4 (A) *Ascl2* KO genotyping by genomic PCR. Wildtype band is 2563 bp and Ascl2 KO band 412 bp. (B) RT-qPCR analysis of formative marker expression in indicated cells in 2iL and after 24h in N2B27. Error bars represent S.D. from technical duplicates.

Fig.S5

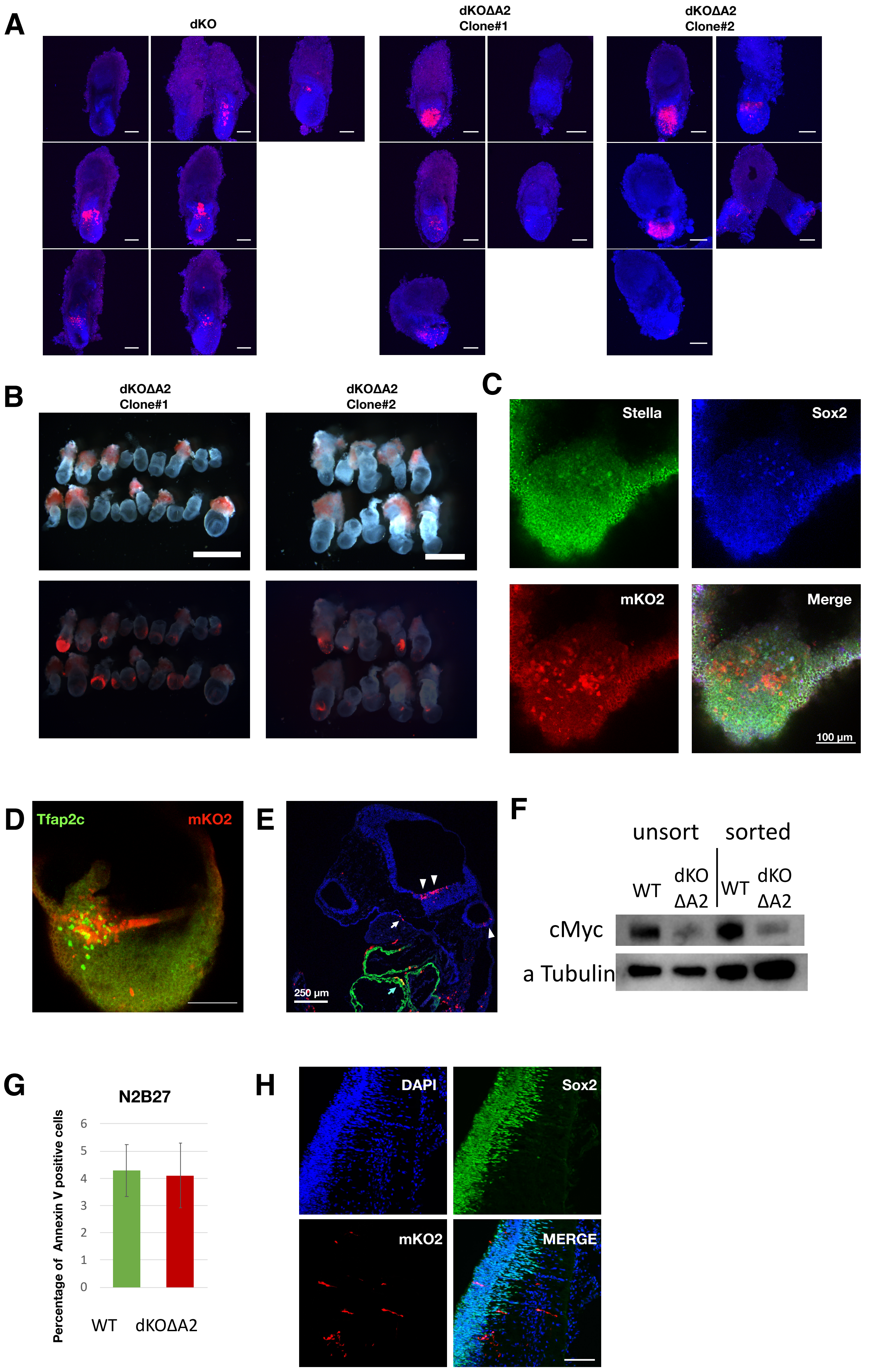
A
dKO
dKOΔA2
Clone#1
dKOΔA2
Clone#2
B
dKOΔA2
Clone#1
dKOΔA2
Clone#2
C
Stella
Sox2
mKO2
Merge
100 μm
D
Tfap2c
mKO2
E
250 μm
F
unsort
sorted
WT
dKO
ΔA2
WT
dKO
ΔA2
cMyc
a Tubulin
G
N2B27
Percentage of Annexin V positive cells
6
5
4
3
2
1
0
WT
dKOΔA2
H
DAPI
Sox2
mKO2
MERGE

Fig. S5 (A) Related to Fig5A. Maximum projection images from z-stack confocal microscopy slices of E6.5 chimaeric embryos. Eomes staining is in blue and mKO2 reporter in red. Scale bar, 100µm. (B) E7.5 chimaeras from two mKO2 reporter Dnmt3dKOΔA2 ES cell lines. Scale bars, 1mm (left), 500µm (right). (C) E7.5 chimaeric embryo immunostained for Stella (green) and Sox2 (blue). Scale bar, 100 µm. (D) E7.5 chimaera immunostained for Tfap2c (green). (E) Section of E9.5 chimaeric embryo immunostained for Sox2 (blue) and cTnT (green). mKO2 (red) positive cells are present in neuroepithelium (white arrowhead), foregut (white arrow) and cardiac mesoderm (blue arrow). Scale bar, 250µm. (F) Western blot analysis of cMyc protein in WT and Dnmt3dKOΔA2 cells. Proteins were collected from independent WT or Dnmt3dKOΔA2 FS cell cultures (unsort) and by sorting GFP positive or mKO2 positive fractions from a mixed culture after 1 day (sorted). (G) Proportions of Annexin V positive cells of each genotype determined by flow cytometry after co-culture for 1 day in N2B27 medium. Error bars represents SD from 6 experiments. P>0.05. (H) Confocal microscope images of Sox2 immunostaining and mKO2 reporter expression in brain region of chimaera shown in Fig 5K. Scale bar, 100µm.

Table S1. List of Primers and gRNAs

Taqman Probes

Gene names	
Actinb	Mm02619580_g1
GAPDH	Mm99999915_g1
Pax6	Mm00443081_m1
Foxa2	Mm01976556_s1
Nanog	Mm02019550_s1
Otx2	Mm00446859_m1
Ascl2	Mm01268891_g1

UPL primers (Used except where SYBR primers are specified)

Gene names	FW	RV	Probe number
Pou5f1	gttggagaaggtggaaccaa	ctccttctgcagggctttc	95
Sox2	ggacttctttttggggact	cagatctatacatggtccgattcc	70
Nanog	ttcttgcttacaagggtctgc	agaggaagggcgaggaga	110
T/Bra	cagcccacctactggctcta	gagcctggggtgatggta	100
Otx2	gacccggtacccagacatc	gctcttcgattcttaaaccatacc	103
Eomes	accggcaccaaactgaga	aagctcaagaaaggaaacatgc	9
Pou3f1	catctccacccgcaagac	cgttcgttaaggccaggag	38
Fgf5	aaaacctggtgcaccctaga	catcacattcccgaattaagc	29
Sox1	gtgacatctgcccccatc	gaggccagtctggtgtcag	60
Dnmt3a	cgcagcgtcacacagaag	gcagggttgacaatggagag	16
Dnmt3b	gtccggaaaatcaccaagaa	cgttagagagatcattgcatgg	31
Sox17	cacaacgcagagctaagcaa	cgcttctctgccaaggtc	97
Mixl1	ccatgtacccagacatccact	cggttctggaaccacacct	71
Ascl2	gagagctaagcccgatgga	aggtccaccaggagtcacc	17
Elf5	gactgtcacagccgaacaag	ccaggatgccacagttctct	56
Tpbpa	tgaagagctgaaccactgga	caggcataggatgactaggaagat	107
Tfap2c	ctgggcagacaagaaactcc	tcgatccctttatctgacgact	62
Hand1	caagcggaaaagggagttg	gtgcgccctttaatcctctt	51
Cdx2	caccatcaggaggaaaagtga	ctgcggttctgaaaccaaat	34
Rhox6	tgggtgtggatgaatgtgat	gcactctcctgtttctctgga	91

SYBR Green Primers

Gene names	FW	RV	
Actinb	ACCAGAGGCATACAGGGACA	ACCAGAGGCATACAGGGACA	Used in Fig. 1D, 1E, S1C, S1D, S1E, S1F, S1H
Ascl2	GCCCGGAGCATGGAAGCACACCTT	TCAGTAGCCCCCTAACCAAGTGGA	Used in Fig. 1D, 1E, S1C, S1D, S1E, S1F, S1H
Elf5	TGAAAACAAGTGGCATCAAGAG	TCAGGGGACAGCAGCAAG	Used in Fig. 1D, 1E, S1E
Tpbpa	CAGGTACTTGAGACATGACTC	GGCAGAGATTTCTTAGACAATG	Used in Fig. 1D, 1E, S1C, S1D, S1E, S1F
Tfap2c	CGCGGAAGAGTATGTTGTTG	TATGTTCGGCTCCAAGACCT	Used in Fig. 1D, 1E, S1E, S1H
Cdx2	CACTTTAGTCGATACATCACCATC	GATTTTCCTCTCCTTGGCTCT	Used in Fig. 1D, 1E, S1E
Hand1	GCGCCTGGGTACCAGTTACA	AGCAACGCCTTCCCTCTAGG	Used in Fig. 1E, S1C, S1D, S1F
PL1	CTCACTTGGAGCCTACATTG	ACCAGGTGTTTCAGAGGTTC	Used in Fig. 1D, S1E
Pou3f1 (Oct6)	TTTCTCAAGTGTCCCAAGCC	ACCACCTCCTTCTCCAGTTG	Used in Fig. 1D, S1E
Otx2	GACCCGGTACCCAGACATC	GCTCTTCGATTCTTAAACCATACC	Used in Fig. 1D, S1E
Pou5f1 (Oct4)	CAGGGTCTCCGATTTGCAT	GCAGCTCAGCCTTAAGAACA	Used in Fig. 1D, S1E, S1L
Nanog	AGGGTGTGCTACTGAGATGCTCTG	CAACCACTGGTTTTTCTGCCACCG	Used in Fig. 1D, S1E

Ascl2 Genotyping primers and gRNAs

Ascl2 gRNA1	gAAGATGTCATTCCGCGGCG
Ascl2 gRNA2	gTTGGCGCGGTCCTATGCCG

Ascl2 genotype F	aggttggaaagttctagacagtgg
Ascl2 genotype R	ctcaagcccttctcacactcaag

Amplicon size WT 2563bp
KO 393 bp

Bisulfite primers

Region 1	Forward	Reverse
Primary primers	GGGTTAAATGGGTATAGGTGTTTAG	TCCTAATACTCAACCCTTAAACCC
Nested Primers	AGGGGTATAGGTGTGTTTTTGTAAT	AAATCTCAACCAATCTAAAATC

Region2		
Primary primers	TTGGATTAGGATGATATAGTTTTGA	ACACACCCCATACCTCTATATTTTC
Nested primers	TTTTGGAGAGTTATGGAATTTGTAG	AAATCTACCCAAACTAATTTTTATTC
